# Differences in DNA Methylation Reprogramming Underlie the Sexual Dimorphism of Behavioral Disorder Caused by Prenatal Stress in Rats

**DOI:** 10.3389/fnins.2020.573107

**Published:** 2020-10-21

**Authors:** Lei Lei, Xinmiao Wu, Hanwen Gu, Muhuo Ji, Jianjun Yang

**Affiliations:** ^1^Department of Anesthesiology, Pain and Perioperative Medicine, The First Affiliated Hospital of Zhengzhou University, Zhengzhou, China; ^2^Department of Anesthesiology, Jinling Hospital, School of Medicine, Nanjing University, Nanjing, China

**Keywords:** DNA methylation, sexual dimorphism, prenatal stress, DNMT, TET

## Abstract

Prenatal stress (PS) can lead to neuroendocrine and emotional disorders later in adolescence. Sexual dimorphism in these neurodevelopmental outcomes have been observed; however, the underlying mechanisms are not fully understood. To address this issue, we investigated whether there are sex differences in epigenetic reprogramming in rats exposed to PS. Pregnant female rats were subjected to chronic restraint stress from gestational day (G)12 to G18. From postnatal day (P)38 to P45, subgroups of offspring including both males and females were subjected to behavioral testing and brain tissue specimens were analyzed by DNA pyrosequencing, western blotting, and Golgi staining to assess changes in methylation pattern of glucocorticoid receptor (GR) gene, expression of DNA methyltransferase (DNMT) and DNA demethylase, and dendrite morphology, respectively. The DNA methyltransferase inhibitor decitabine was administered to rats prior to PS to further evaluate the role of methylation in the sexually dimorphic effects of PS. The results showed that PS increased anxiety-like behavior in offspring, especially in females, while depression-like behavior was increased in male offspring compared to control littermates. The methylation pattern in the promoter region of the GR gene differed between males and females. Sex-specific changes in the expression of DNMTs (DNMT1 and DNMT3a) and DNA demethylase (Tet methylcytosine dioxygenase 2) were also observed. Interestingly, decitabine alleviated the behavioral disorder caused by PS and restored dendrite density and morphology in female but not male rats. These findings suggest that different change patterns of DNMT and demethylase in the two sexes after PS are responsible for the sexually dimorphism, which could have implications for the clinical management of stress-related disorders.

## Introduction

Exposure to adverse stimuli during gestation can have persistent effects on cognition and emotion in adults. Specific outcomes vary depending on the nature, intensity, and timing of exposure as well as species, sex, and age (Hamada and Matthews, 2019). Multiple psychological disorders associated with PS have been reported in humans and animal models including depression, anxiety, and attention deficit hyperactivity disorder (ADHD) (Weinstock, 2001; Walsh et al., 2019; Scheinost et al., 2020), which have in common a dysregulation of the stress response system (Ramocki and Zoghbi, 2008). Glucocorticoids can influence neural circuits in the developing brain at the time of stress exposure, which can have long-term effects on brain structure, function, and plasticity (Kinlein et al., 2019). Interestingly, these effects were shown to be sexually dimorphic (Schulz et al., 2011; Iturramena et al., 2018). For example, exposure to social stress during gestation resulted in elevated anxiety in male but not female rats (García-Cáceres et al., 2010). Normal hypothalamic development differs between the sexes, resulting in sexual dimorphism in the structure and function of the hypothalamus; therefore, environmental challenges during this critical period can have sex-specific effects, although the molecular basis is not well understood (Hodes and Epperson, 2019).

A possible mechanism underlying the persistent effects of early life stresses is epigenetic reprogramming, which is responsible for cell type-specific and experience-dependent changes in gene expression during embryonic and postnatal development (Szyf, 2019). DNA methylation, which is the covalent modification of DNA with a methyl group, is among the most extensively studied epigenetic events and has been implicated in the genomic encoding of early-life experiences, and is thought to link environmental stimuli to behavioral phenotypes (Auger and Auger, 2013). Sex differences have been reported in the DNA methylation patterns of promoter regions of steroid hormone receptor genes. We speculated that this contributes to the sexual dimorphism of brain structures and provides a physical substrate for the sex-specific behavioral effects of PS.

To test the hypothesis, we investigated the methylation of GR gene in rats exposed to PS, and tested whether different change patterns of DNMT and demethylase involved in sexual dimorphism of behavioral disorders and dendritic spine abnormalities caused by PS.

## Materials and Methods

### Animals and Treatment Groups

Sprague-Dawley rats were housed in a temperature-controlled room (20°C) on a 12:12 h light/dark cycle with free access to food and water. Female rats were mated with males for 1 night and vaginal smears were examined the next day; the presence of sperm was taken as pregnancy. The day after mating was recorded as gestational day (G)1 and pregnant rats were individually housed in the same environment until G11. Prior to restraint stress, the rats were randomly divided into control, stress (PS), decitabine, and vehicle groups. The last three groups were subjected to restraint stress from G12 to G18 while control rats were left undisturbed. Decitabine group and vehicle group were treated with decitabine [0.5 mg/kg by intraperitoneal injection (i.p.)] or vehicle (10% dimethyl sulfoxide, i.p.) for 3 consecutive days (G12–G14) 30 min prior to PS, respectively. The dose of 0.5 mg/kg was selected based on previous reports (Shen et al., 2019). Litter size at birth ranged from 7 to 17 pups, and on postnatal day (P)2, litters were culled to 10 pups for a male-to-female ratio of 1 in each litter wherever possible. At P22, pups were distributed (*n* = 2/cage) according to their origin from control or stressed dams, with males and females housed separately. The pups were used for behavioral experiments at around P45 (equivalent to adolescence). All experimental procedures were conducted in accordance with the guidelines of the Institutional Animal Care and Use Committee of Zhengzhou University.

### Chronic Restraint Stress (CRS)

Pregnant rats were subjected to CRS from G12 to G18 for 3 sessions per day (45 min per session starting at 09:00, 11:00, and 13:00 h), during which they were placed in transparent plastic cylinders (inner diameter: 9 cm, length: 19 cm) and exposed to bright light (Marrocco et al., 2014). The control group was left undisturbed in their home cage. Tail blood samples (∼300 μl) were collected before the first CRS session at G12 and 15 min after the last session at G18.

### Behavioral Tests

A well-trained investigator who was blinded to animal grouping performed the behavioral tests including the open field test (OFT at P45), elevated plus maze (EPM at P46), sucrose preference test (SPT at P47), and forced swim test (FST at P50) as we described previously (Ji et al., 2020). Behavioral testing was carried out in the same order for all rats during the light phase of the circadian cycle (09:30–14:00 h).

#### OFT

Rats were placed in the center of the arena (100 × 100 × 40 cm) and allowed to explore the open field for 5 min, and the total distance moved was recorded using Panlab SMART 3.0 video tracking software (RWD, Shenzhen, China). The chamber was cleaned with 75% ethanol after each test.

#### EPM

The EPM consisted of 2 opposing open arms (50 × 10 × 0.5 cm) and 2 perpendicular opposing arms (50 × 10 × 45 cm) elevated 75 cm above the floor. Testing was carried out in a dimly lit room with a 40 W bulb hung 60 cm above the central part of the maze. Rats were placed in the center square facing an open arm and were allowed to explore the maze for 5 min. During the testing period, the rat’s behavior was recorded using Panlab SMART 3.0 video tracking software (RWD, Shenzhen, China). The number of open arms entries and time spent in the open arms were noted. If a fall occurred, the animal was removed from the study.

#### SPT

Beginning at P47, rats were habituated to drinking a solution of 1% sucrose for 24 h. At P48, control and PS rats were given the choice between a bottle containing normal drinking water and another containing sucrose solution. The bottles were left for 24 h, with their positions switched after the first 12 h. At P49, each rat was given 2 pre-quantified bottles, one containing normal drinking water and the other containing sucrose solution. The test lasted 24 h, with the positions of the 2 bottles switched after the first 12 h. After 24 h, the bottles were removed and weighed. Sucrose preference was calculated as the percentage of sucrose intake relative to total fluid intake (water + sucrose).

#### FST

Rats were placed in a plastic cylinder (diameter: 50 cm, height: 80 cm) filled with water (22–25°C) for 6 min, and immobility time in the final 5 min was recorded. The water was changed between tests so that fresh water was used for each animal. Immobility, which was defined as the absence of movement except for leg kicks to stay afloat, was used as a measure of behavioral despair and helplessness (i.e., depression-like behavior). After the test, rats were removed from the water, dried with towels, and sacrificed the next day under isoflurane anesthesia.

### Western Blotting

Expression levels of glucocorticoid receptor (GR), the DNMTs DNMT1 and DNMT3a, and the DNA demethylase Tet methylcytosine dioxygenase (Tet)-2 in the hippocampus were evaluated by western blotting. Total protein was extracted from hippocampal tissue samples and quantified, and equal amounts were loaded and separated by sodium dodecyl sulfate polyacrylamide gel electrophoresis and transferred to a nitrocellulose membrane (Millipore, Billerica, MA, United States) that was blocked in 3% non-fat milk for 1 h at room temperature followed by overnight incubation at 4°C with primary antibodies against GR (Cell Signaling Technology, Danvers, MA, United States; 12041S, 1:1,000), DNMT1 (ab188453, 1:1,000) and DNMT3a (ab188470, 1:1,000) (both from Abcam, Cambridge, MA, United States), Tet-2 (ABclonal, Woburn, MA, United States; A1526, 1:1,000), and β-actin (Proteintech, Chicago, IL, United States; 66,009–1, 1:2,000). The specificity of antibodies was tested using western blotting, which showed that only one band was detected at the expected molecular weight in all antibodies. The membrane was then incubated with appropriate secondary antibodies conjugated with horseradish peroxidase (1:10,000; Abbkine, Beijing, China) for 2 h at room temperature. Protein bands were detected using the ChemiDoc MP system (Bio-Rad, Hercules, CA, United States).

### Enzyme-Linked Immunosorbent Assay (ELISA)

Blood samples were collected and centrifuged at 3,000 rpm for 15 min. Serum cortisol (CORT) was measured in duplicate using a commercially available enzyme immunoassay kit (Cayman Chemical Company, Ann Arbor, MI) according to the manufacturer’s instructions.

### Golgi Staining

Rats were anesthetized with isoflurane and sacrificed by decapitation. The whole brain was removed from the skull and stained using the FD Rapid Golgi stain Kit (FD NeuroTechnologies, Columbia, MD, United States; PK401) as we previously described (Hao et al., 2015). Specially, there were 5 rats per group and about six neurons from brain slices of the same coronal section in each rat were chosen. Next, three segments of dendrites from each neuron were selected, and the average value of each 50 μm dendritic spine was calculated.

### DNA Pyrosequencing

DNA was extracted from hippocampal tissue samples using the DNeasy Blood and Tissue kit (Qiagen, Hilden, Germany) according to the manufacturer’s instructions, and sodium bisulfite modification was performed using the Qiagen EpiTect Bisulfite Kit (Qiagen). Primers (forward: TTTTAATTAGGGATTTTTAAGAGGTTAGGT; reverse: CCACAAATACCAACCCTTAACACTTCTAAT) for amplifying the bisulfite-converted DNA sequences were designed using PyroMark Assay Design v2.0 software (Qiagen). PCR amplification was performed on an ABI 9700 PCR system (Applied Biosystems, Foster City, CA, United States). Pyrosequencing and analysis of DNA methylation status of all CpG sites were performed using PyroMark Q96 ID (Qiagen).

### Statistical Analysis

Statistical analyses were performed using SPSS v20.0 (SPSS Inc., Chicago IL, United States) and Prism v7.0 software (GraphPad, San Diego, CA, United States). Data are presented as mean ± SEM. Two-way analysis of variance (ANOVA) was used to assess body weight gain in dams, differences in CORT concentration and effects of treatments and sex. *T*-test and one-way ANOVA were used to assess behavioral results and other data. For comparison of two groups, we used unpaired *t*-test for normally distributed data and Mann-Whitney test for other data. For comparisons of multiple groups, we applied one-way ANOVA followed by Bonferroni *post hoc* test for normally distributed data and non-parametric Kruskal-Wallis test followed by Dunn’s test for data with other types of distributions. *P* < 0.05 was considered statistically significant in all tests.

## Results

### Effect of PS on Intrauterine Development

**FIGURE 1:**
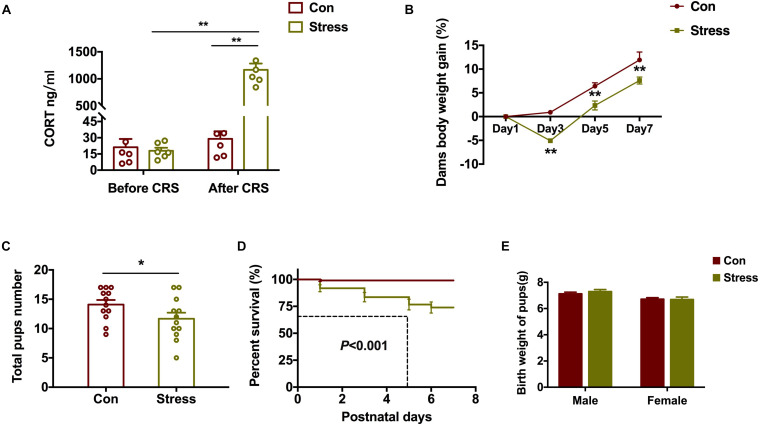
Effect of PS on embryo development. **(A)** Stressed dams showed increased serum CORT levels after CRS (**P < 0.01, *n* = 6). **(B–D)** Compared to control dams, those subjected to CRS showed less body weight gain (B) (**P < 0.01, *n* = 8–11) and gave birth to fewer pups **(C)** (*P < 0.05, *n* = 12) that had a lower survival rate **(D)** (**P < 0.01, *n* = 12). **(E)** PS did not affect the birth weight of pups (*n* = 38). *P < 0.05, *P < 0.01 vs. corresponding control group. Data represent mean ± SEM. Con, control.

To evaluate the effects of PS on embryo development, we measured serum CORT before (G12) and after (G18) CRS by ELISA. Serum CORT levels increased markedly after CRS [Groups: *F*(1, 20) = 94.36, *P* < 0.001; CRS: *F*(1, 20) = 99.06, *P* < 0.001; Figure 1A]. Stressed dams gained less weight starting from day 3 of CRS [Groups: *F*(1, 68) = 35.62, *P* < 0.001; Days: *F*(3, 68) = 75.77, *P* < 0.001; Figure 1B]. Additionally, compared to control rats, the total number of pups born [*t*(22) = 2.25, *P* = 0.035; Figure 1C] and pup survival rate [χ^2^(1) = 25.96, *P* < 0.001; Figure 1D] were lower in stressed dams, although there was no significant difference in the birth weight of the pups (Figure 1E). These results indicate that PS induces a stress response in dams that has a negative effect on the viability of rat embryos.

### Effect of PS on Behavior in Adolescence

**FIGURE 2:**
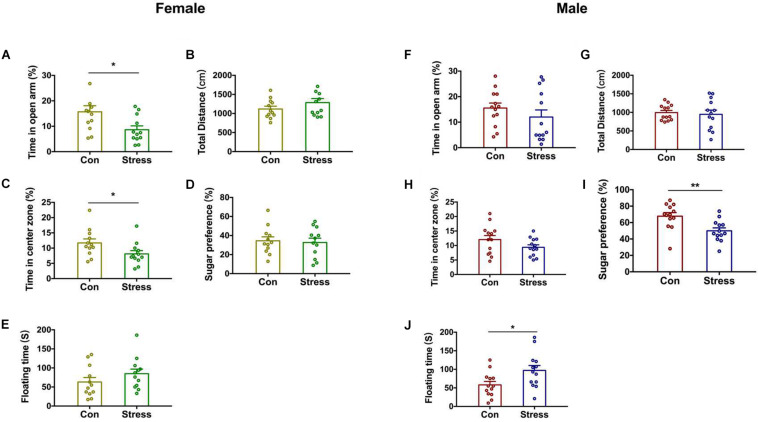
Effect of PS on the behavior of adolescent rats. **(A)** PS females spent less time in the open arms of the EPM than control females (*P < 0.05, *n* = 12). **(B)** Total distance traveled was similar in PS and control females. **(C)** PS females spent less time in the center zone in the OFT than control females (*P < 0.05, *n* = 12). **(D,E)** PS did not affect depression-like behavior of females. **(F–H)** PS did not affect anxiety-like behavior in males. **(I,J)** PS reduced sugar preference in the SPT (I) (**P < 0.01, *n* = 13) and increased floating time in the FST **(J)** (*P < 0.05, *n* = 13) compared to the control group in male rats. *P < 0.05, **P < 0.01 vs. corresponding control group. Data represent mean ± SEM. Con, control.

Stress is a major contributor to the etiology of anxiety and depression (Dedic et al., 2019). We used the EPM and OFT to evaluate the effects of CRS on anxiety-like behavior in pups in later life. Notably, PS female rats showed a higher degree of anxiety-like behavior than control female rats, spending less time in the open arms of the EPM [*t*(22) = 2.438, *P* = 0.021; Figure 2A] and in the center zone in the OFT [*t*(22) = 2.104, *P* = 0.047; Figure 2C]. On the other hand, total distance traveled did not differ between the 2 groups (Figure 2B), implying that locomotor behavior was unaffected by PS. CRS had no effect on anxiety-like behavior in males (Figures 2F,H). We next examined whether PS affects depression-like behavior in offspring with the SPT and FST (Figures 2I,J) and found that sugar preference was reduced [*t*(24) = 2.881, *P* = 0.008; Figure 2G] and floating time was increased [*t*(24) = 2.429, *P* = 0.023; Figure 2H] by CRS in male but not female rats (Figures 2D,E). Two-way ANOVA analysis further revealed significant effects of treatments and sex on SPT [Treatments: *F*(1, 46) = 5.597, *P* = 0.022; Sex: *F*(1, 46) = 37.15, *P* < 0.001]. These results indicate that the effects of PS on behavior in adolescence are sex-specific.

### Effect of PS on Epigenetic Modification of the GR Gene in Adolescence

**FIGURE 3:**
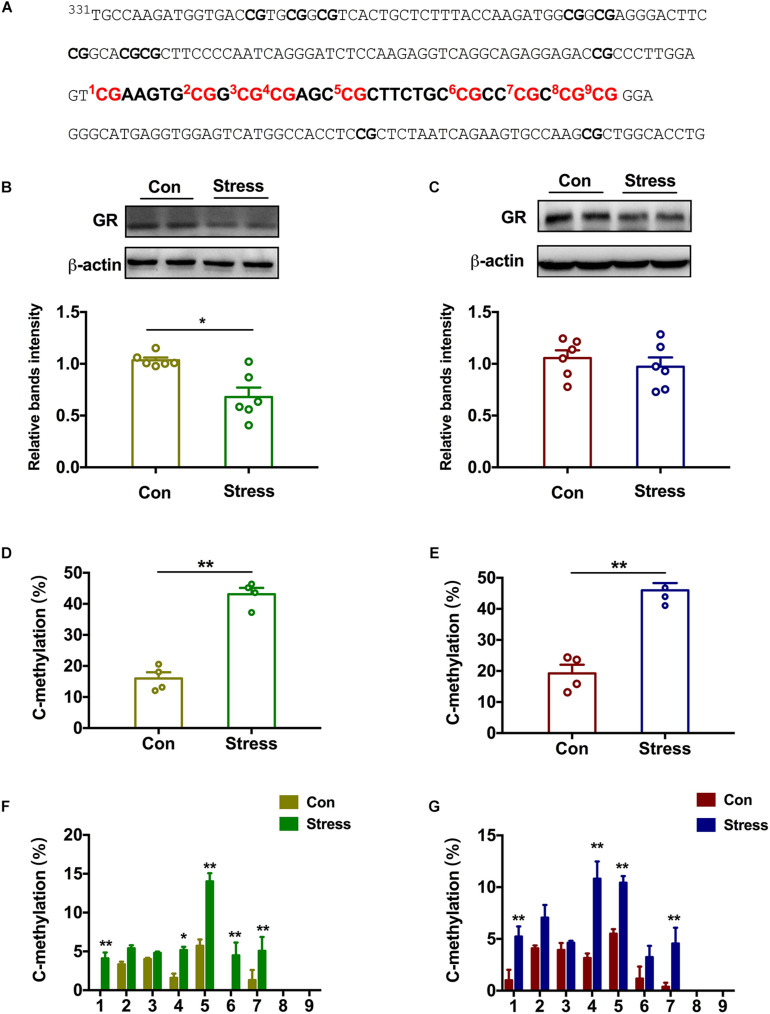
Effect of PS on epigenetic modification of the GR gene in adolescent rats. **(A)** Sequence of the promoter region of the GR gene *Nr3c1* containing 9 CpG dinucleotides. **(B,C)** GR protein level in the hippocampus was reduced in PS females **(B)** (*P < 0.05, *n* = 6) but not males **(C)**. **(D,E)** PS females **(D)** and males **(E)** had a higher percentage of methylated sites than the respective controls (**P < 0.01, *n* = 4). **(F)** All 9 CpG sites except for sites 2, 3, 8, and 9 showed increased methylation in PS females compared to control females (**P < 0.01, *n* = 4). **(G)** CpG sites 1, 4, 5, and 7 showed increased methylation in PS males compared to control males (**P < 0.01, *n* = 4). *P < 0.05, *P < 0.01 vs. corresponding control group. Data represent mean ± SEM.

The hippocampus is the negative feedback center of the hypothalamic–pituitary–adrenal (HPA) axis and has the highest expression of GR (Antunes et al., 2020). We examined GR protein level in the hippocampus of adolescent rats and found that it was decreased in PS females [*t*(10) = 3.728, *P* = 0.015; Figure 3B] but not PS males (Figure 3C) relative to the respective controls. Two-way ANOVA also revealed significant effects of treatments and sex on the expression of GR [Treatments: *F*(1, 20) = 8.379, *P* = 0.009; Sex: *F*(1, 20) = 4.786, *P* = 0.041]. To clarify the mechanistic basis of this observation, we performed DNA pyrosequencing to examine the methylation status of 9 CpGs in the promoter of the GR gene *Nr3c1* in hippocampal tissue samples from P45 offspring (Figure 3A). Total methylation frequency of CpG sites differed significantly both in PS females [*t*(6) = 9.472, *P* < 0.001; Figure 3D] and PS males [*t*(6) = 7.32, *P* < 0.001; Figure 3E) relative to the respective controls. Specially, methylation patterns at all 9 CpG sites expect for sites 2, 3, 8, and 9 were altered in females [Groups: *F*(1,54) = 68.68, *P* < 0.001; Sites: *F*(8, 54) = 29.43, *P* < 0.001; Figure 3F] whereas in males, CpG sites 1, 4, 5, and 7 showed increased methylation as a result of PS exposure [Groups: *F*(1, 54) = 56.46, *P* = 0.004; Sites: *F*(8, 54) = 22.89, *P* < 0.001; Figure 3G]. Thus, PS induced different expression of GR in the hippocampus of adolescent rats between the two sexes, which was accompanied by a change in methylation of the gene promoter.

### Effect of PS on DNMT and Demethylase in Adolescence

**FIGURE 4:**
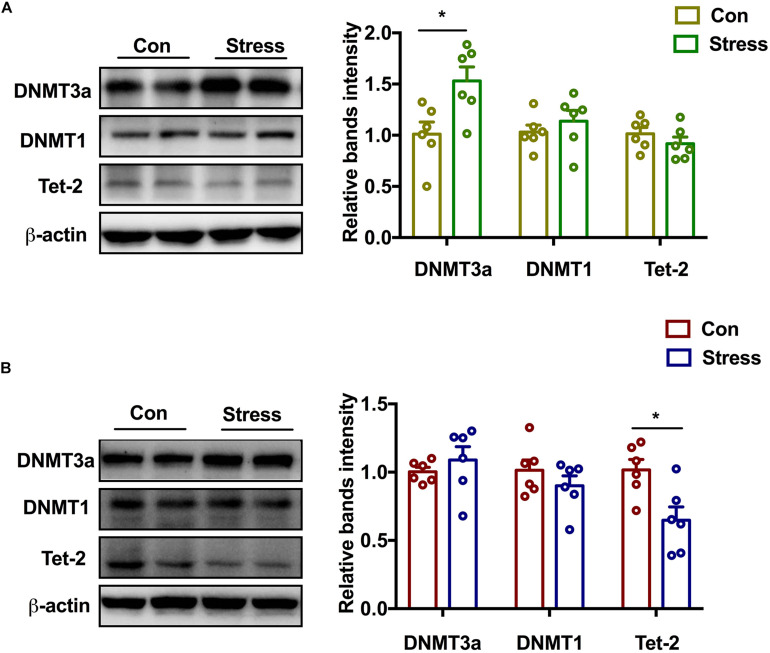
Effect of PS on DNMT and demethylase in adolescence. **(A)** DNMT3a protein expression was markedly upregulated (*P < 0.05, *n* = 6) while DNMT1 level was marginally increased in PS females. No significant difference in Tet-2 expression was observed between PS and control females. **(B)** Tet-2 level was decreased in PS males (*P < 0.05, *n* = 6), whereas DNMT3a and DNMT1 levels were similar in PS and control males. *P < 0.05, *P < 0.01 vs. corresponding control group. Data represent mean ± SEM.

To elucidate the upstream regulator of epigenetic reprogramming in PS offspring, we examined which DNMT or DNA demethylase is involved in the hypermethylation of GR. PS exposure markedly increased DNMT3a [*t*(6) = 2.861, *P* = 0.017] and marginally increased DNMT1 protein levels in the hippocampus of adolescent female rats, although no changes were observed in Tet-2 expression (Figure 4A). In male rats, Tet-2 was downregulated following PS exposure [*t*(10) = 2.975, *P* = 0.014] whereas DNMT3a and DNMT1 expression was unchanged relative to the control group (Figure 4B). Two-way ANOVA also revealed significant effects of treatments and sex on the expression of DNMT3a [Treatments: *F*(1, 20) = 8.38, *P* = 0.009; Sex: *F*(1, 20) = 4.631, *P* = 0.044]. Thus, PS induces sex-specific GR reprogramming by differentially modulating the expression of DNMT and DNA demethylase in male and female offspring.

### DMNT Inhibition Restores Normal Behavior and Dendrite Plasticity in Rats Exposed to PS

**FIGURE 5:**
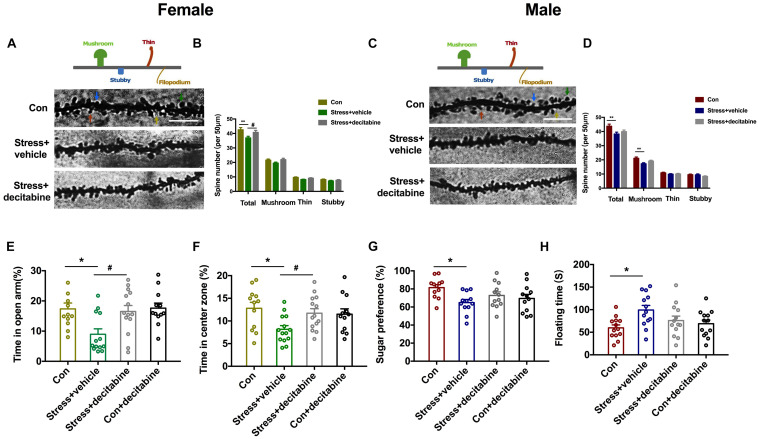
DNMT inhibition restores normal behavior and dendritic spine density in PS-exposed rats. **(A,C)** Representative image of dendritic spines of pyramidal neurons in the hippocampal CA1 area of adolescent female **(A)** and male **(C)** rats (scale bar = 10 μm). **(B,D)** Quantitative analysis of spine density. The density was decreased in PS females treated with the vehicle dimethylsulfoxide (**P < 0.01, *n* = 30); decitabine restored spine density (*P < 0.05, *n* = 30) **(B)**. In male PS rats treated with vehicle, the density of dendritic spines (**P < 0.01, *n* = 30), especially mushroom spines (**P < 0.01, *n* = 30), was decreased; decitabine did not rescue the decrease in spine density or restore subtype ratios **(D)**. **(E,F)** Female rats injected with vehicle spent less time in the open arms of the EPM (E) (*P < 0.05, *n* = 12–14) and center zone in the OFT **(F)** (*P < 0.05, *n* = 12–15) than rats in the decitabine group. **(G,H)** Sucrose preference in the SPT **(G)** and floating time in the FST **(H)** were similar in PS males compared to control males. *P < 0.05, **P < 0.01 vs. corresponding control group; ^#^*P* < 0.05 vs. Stress/vehicle group. Data represent mean ± SEM. Con, control.

To further investigate the role of DNA methylase in the neurodevelopmental outcomes of PS, we examined whether the DNMT inhibitor decitabine could affect the behavior and dendrite plasticity of PS offspring. At P45, dendritic spine density of pyramidal neurons in the hippocampal CA1 area was evaluated by Golgi staining. The spine density [Females: *F*(2, 86) = 6.441, *P* = 0.003, Figures 5A,B; Males: *F*(2, 86) = 4.739, *P* = 0.011, Figures 5C,D] was significantly lower in PS rats (Females: *P* = 0.002 vs. control group; Males: *P* = 0.007 vs. control group) compared to control rats; decitabine partly reversed this decrease, but only in females (*P* = 0.022 vs. stress group; Figures 5B,D). We further examined spine subtypes [Males: *F*(2, 86) = 6.387, *P* = 0.003] and found that the ratio of mushroom spines to stubby and thin spines was only reduced in males (*P* = 0.002 vs. stress group; Figure 5D), which was not rescued by treatment with decitabine.

We next examined whether decitabine could restore normal behavior in PS-exposed rats. Female rats in the PS group that were treated with decitabine spent a longer time in the open arms of the EPM [*F*(3, 47) = 4.942, *P* = 0.005, *P* = 0.025 vs. stress/vehicle group; Figure 5E] and in the open area in the OFT [*F*(3, 49) = 3.772, *P* = 0.016, *P* = 0.047 vs. stress/vehicle group; Figure 5F] than those treated with vehicle. Besides, decitabine did not affect the behavior performance of control females. Interestingly, the protective effects of decitabine were not observed in male offspring. Decitabine did not rescue depression-like behavior in the SPT and FST (Figures 5G,H). Taken together, our results suggest that neurodevelopmental deficits in female but not male rats exposed to PS might be DNA methylation-dependent.

## Discussion

The present study provides evidence that PS exposure induced increased anxiety-like behavior in female offspring and depression-like behavior in male offspring, respectively. Moreover, the methylation pattern in the promoter region of the GR was sex-specific, accompanied by changes in DNMTs and DNA demethylase. Interestingly, decitabine alleviated the behavioral disorder caused by PS and restored dendrite density and morphology in female but not male rats. These findings suggest that different change patterns of DNMT and demethylase in the two sexes after PS are responsible for the sexual dimorphism.

PS can critically alter neurophysiology and behavior in offspring, with long-term consequences. The increased maternal plasma levels of CORT—which can cross the placental barrier—caused by stress have been shown to inhibit embryo growth and reduce birth weight (Iturramena et al., 2018). It is possible that PS-exposed dams in our study gained less weight than controls dams; however, there was no difference in the birth weights of pups, implying that some long-term changes had already occurred in the embryo.

Recent studies have focused on sex differences in psychopathologic outcomes associated with PS including anxiety, depression, ADHD, schizophrenia, and autism (Weinstock, 2001; Walsh et al., 2019; Davies et al., 2020; Scheinost et al., 2020). Chronic maternal stress was shown to increase anxiety- and depression-like behavior in prepubertal males relative to controls, whereas prepubertal females were less affected (Schulz et al., 2011). Another study in rats found that predator odor stress during the second half of pregnancy enhanced the anxiety-like phenotype in adult male and female offspring and increased defensive behavior in males (St-Cyr et al., 2017). We showed here that PS increased in anxiety-like behavior in females rats whereas depression-like behavior was evident only in males. Given that both sexes were similarly exposed to high levels of maternal CORT in the intrauterine environment, it is unclear how such sexually dimorphic outcomes arose. The fetal HPA axis and limbic brain areas regulating the axis such as the hippocampus mature late in gestation (Wood and Walker, 2015). Overexposure of the fetal brain to maternal CORT increases negative feedback control of the axis to counter the effects of elevated maternal corticosterone (Krontira et al., 2020). PS was shown to decrease GR expression in the amygdala and hippocampus to a greater extent in the female fetus than in the male fetus (Lan et al., 2017). This is consistent with our finding that hippocampal GR expression was downregulated in PS females. GR plays a key role in promoting resilience by counteracting the genomic effects of CORT overexposure in the brain of stressed animals (Han et al., 2017); our results suggest that the brain of the female fetus is more resilient to CORT overexposure than that of males.

Studies in humans and animal models have revealed aberrations in dendritic spine architecture in several psychiatric disorders including depression and others related to stress (Edfawy et al., 2019; Guo et al., 2019; Yang et al., 2020). Changes in spine density and organization are thought to contribute to the behavioral disorder caused by stress exposure (Chen et al., 2020). Dendrite atrophy in neurons of the prefrontal cortex has been reported in adult animals following stress exposure, and gestational stress between G15 and G20 reduced the density of spines on apical and basal dendrites of neurons in the anterior cingulate cortex by 21% (Meena et al., 2006; Rincel et al., 2018). In this study, PS caused a decrease in spine density—especially of mushroom spines—specifically in adolescent males. In rodents, the reduction in synapse density was preceded by changes in spine morphology, including a reduced length and enlargement of the neck (Androuin et al., 2018). Mathematical modeling indicated that this could alter electrical compartmentalization, leading to reduced postsynaptic potentials in spine heads but not in the soma, thereby impairing long-term potentiation without altering basal synaptic transmission (Weinhard et al., 2018; Washburn et al., 2020). Thus, it may be possible to predict developmental outcomes of early life stress by analyzing the density and morphology of spines in pyramidal neurons.

The persistence of phenotypes resulting from early life stress may be mediated by epigenetic mechanisms. Exposure of human hippocampal neuron progenitors to glucocorticoids caused long-lasting changes in DNA methylation profile (Provençal et al., 2019). In the present study, we showed by bisulfite sequencing that PS exposure increased methylation of CpG sites in the promoter of the GR gene *Nr3c1* in the hippocampus of both sexes. However, the sex-specific expression patterns of GR protein in PS offspring suggest that additional mechanisms are involved. Specific DNMTs or demethylase were found to be involved in anxiety-like behavior in adult rodents; it was suggested that these enzymes exert their effects by modifying GR expression in the hippocampus (Cheng et al., 2018; Ye et al., 2018). In support of this possibility, we showed that PS exposure increased DNMT3a expression in the hippocampus of adolescent female rats while decreasing that of Tet-2 in males. This could explain the inconsistency between GR protein expression and hypermethylation of CpG sites in the *Nr3c1* gene promoter. Bisulfite sequencing cannot distinguish between DNA methylation and hydroxymethylation of CpG sites, which are mediated by DNMT and TET, respectively (Rauluseviciute et al., 2019). It has been reported that hydroxymethylation but not methylation of GR is more prominent in males (Gontier et al., 2018). In the present work, the protective effects of the DNMT inhibitor decitabine further demonstrated that the neurodevelopmental abnormalities caused by PS in female but not male rats are DNA methylation-dependent. However, additional studies are needed to clarify the role of hydroxymethylation in the effects of PS exposure. An ongoing study in our laboratory involving genome-wide analyses of DNA methylation and RNA sequencing will provide information on how PS-induced changes in DNA methylation in specific genomic regions, and how this can be reversed by pharmacologic targeting of DNMTs and DNA demethylase.

Our study has several limitations. First, PS performed in animals ranges from physical stress to psychological stress or both and different PS paradigms make direct comparisons between studies rather difficult. It should be admitted that CRS imposed in the current study is a serious stressor, and our results were somewhat stress-specific and might not be comparable to lighter stressors. Secondly, some confound factors that impact behavior in the FST received little consideration, i.e., age, metabolism, weight, and the ability to stay afloat. In addition, besides the GR in the hippocampus, several other high-order regulators of the HPA axis may also contribute to the changes observed here. Lastly, although decitabine showed therapeutic effects only in female rats, it could not been confirmed that the neurodevelopmental defects in female rats exposed to PS are DNA methylation-dependent. Specifically designed studies will be needed to clarify the role of DNMTs and DNA demethylase and underlying mechanisms.

In summary, the results presented here provide novel insight into the molecular mechanisms underlying sex differences in neuroanatomy and behavior caused by prenatal stress exposure, with the evidence suggesting that DNMT and demethylase reprogramming is involved. These findings can serve as a basis for the development of improved treatments for psychological disorders associated with PS.

## Data Availability Statement

All datasets presented in this study are included in the article/supplementary material.

## Ethics Statement

The animal study was reviewed and approved by the Institutional Animal Care and Use Committee of Zhengzhou University.

## Author Contributions

JY designed the study. LL, XW, HG, and MJ performed the experiments. LL and XW analyzed the data. LL, MJ, and JY wrote the manuscript. All authors contributed to the article and approved the submitted version.

## Conflict of Interest

The authors declare that the research was conducted in the absence of any commercial or financial relationships that could be construed as a potential conflict of interest.
